# Development and psychometric properties of short form of central sensitization inventory in participants with musculoskeletal pain: A cross-sectional study

**DOI:** 10.1371/journal.pone.0200152

**Published:** 2018-07-05

**Authors:** Tomohiko Nishigami, Katsuyoshi Tanaka, Akira Mibu, Masahiro Manfuku, Satoko Yono, Akihito Tanabe

**Affiliations:** 1 Department of Nursing and Physical Therapy, Konan Woman’s University, Kobe, Hyogo, Japan; 2 Department of Rehabilitation, Tanabe Orthopaedics, Osaka, Osaka, Japan; 3 Department of Rehabilitation, Breast Care Sensyu Clinic, Osaka, Kishiwada, Japan; Parc Sanitari Sant Joan de Déu, SPAIN

## Abstract

**Background:**

The central sensitization inventory (CSI) comprises 25 items and is commonly used to measure somatic and emotional symptoms related to central sensitization symptoms. CSI was developed as an easy-to-administer screening instrument for patients at high risk of developing central sensitization in whom it was essential to quickly evaluate the condition. The purpose of the present study was to develop a short form of CSI and evaluate its psychometric properties using a contemporary approach called Rasch analysis.

**Methods:**

A total of 505 patients with musculoskeletal disorders were recruited in this study. The CSI, pain intensity, pain interference, and the health-related quality of life (QOL) were evaluated for each participant. The original CSI items were consecutively analyzed using the Rasch model. Successive Rasch analyses were performed until a final set of items satisfied the model fit requirements. We also analyzed the psychometric properties of the original and short forms of CSI.

**Results:**

Four consecutive Rasch analyses identified the removable items. Finally, the shortest questionnaire obtained that maintained the correct psychometric properties based on the Rasch model contained only 9 items (CSI-9). Rasch analysis showed that the CSI-9 had acceptable internal consistency, exhibited unidimensionality, had no notable differential item functioning, and was functional on the category rating scale.

**Conclusions:**

The nine-item short form of CSI has acceptable psychometric properties and is suitable for use for patients with musculoskeletal pain. Thus, the CSI-9 can be used as a brief instrument to evaluate central sensitization.

## Introduction

The International Association for the Study of Pain defines central sensitization (CS) as an increased responsiveness of nociceptive neurons in the central nervous system to normal or subthreshold afferent input [1]. Several systematic reviews demonstrate that CS plays a significant role in the treatment of patients with osteoarthritis [2], shoulder pain [3], whiplash [4], fibromyalgia [5], and tendinopathy [6]. The Central Sensitization Inventory (CSI) was developed as a screening instrument for CS-related symptoms [7]. The scale comprises 25 items related to the assessment of health-related symptoms that are common to central sensitivity syndromes (CSS), such as restless leg syndrome, chronic fatigue syndrome, fibromyalgia, and temporomandibular joint disorder. Confirmatory factor analysis revealed that a bifactor mode containing one general factor and four orthogonal factors (physical symptoms, emotional distress, headache/jaw symptoms, and urological symptoms) fits the CSI structure better than the unidimensional and the 4-factor models [8].

The CSI total scores have been shown to distinguish between patients with chronic pain and control subjects [9, 10]. A higher CSI score is associated with pain-related outcomes [10, 11] and increase in levels of brain-derived neurotrophic factor [12], which contributes to both induction and maintenance of CS [13], and predicts poor long-term postoperative outcomes [14,15].

CSI has been translated into many languages and has been validated by a variety of practitioners[12, 16–21]. A systematic review revealed that CSI has strong psychometric properties and therefore could be a clinically useful measurement instrument [22]. While CSI comprises 25 items and can be used as a psychometric instrument, it is easier to use in clinical settings it if it is shorter. Alternative questionnaires such as a shorter version of pain catastrophizing scale [23–25], pain beliefs and coping strategies [26–28], and pain-related self-efficacy [29] were developed to facilitate a quick screening and to reduce the participant’s burden. This short version of the questionnaire is also preferable because many health professionals have limited time with patients in a clinical setting. CSI was developed as an easy-to-administer screening instrument for patients at higher risk of developing CS [7] in whom it was essential to quickly evaluate the condition. Thus, a brief questionnaire on CSI has the potential to be clinically useful, to be time efficient, and to reduce patient burden in both clinical and research environments. However, it has been noted that shorter questionnaires risk sacrificing reliability. Therefore, short-form measures must be shown to have an acceptable level of reliability and validity.

The Rasch model [30], which is included in the item response theory, can be used to estimate the item or ability parameters and is a way to analyze responses to questionnaires with the goal of improving measurement accuracy and reliability. It is often used to reduce the items covered in questionnaires [31–36]. This model constructs a line of measurement with the items placed hierarchically [37, 38], which permits identification of redundant items to be omitted from the original questionnaire. The present study aimed to develop a short form of CSI and evaluate its psychometric properties.

## Methods

### Participants

Individuals aged between 20 and 80 years were consecutively recruited and screened by orthopedists to receive physical therapy from an orthopedic clinic. Those with acute (pain that lasts less than 3 months) or chronic (pain that lasts for at least 3 month) musculoskeletal pain disorders (lower back pain, neck pain, shoulder pain, knee pain, ankle pain, and/or hand pain) were included. All participants underwent X-ray examination before receiving physical therapy. At the screening stage by orthopedists, participants with fracture, sprain, cancer, multiple sclerosis, brain or spinal cord injury, history of stroke, or history of psychiatric disease (e.g., schizophrenia, bipolar disorder, or somatoform disorder) as diagnosed by a psychiatrist were excluded.

All evaluations were performed before physical therapy. Of the initial sample of 510 participants, 5 participants were excluded because they did not complete all the items in the questionnaires. A total of 505 individuals were included; all these patients were Japanese. Their characteristics are summarized in Table 1. Patients with musculoskeletal disorders were distributed as follows: 187 patients (37.0%) with lower back pain, 89 (17.6%) with neck pain, 84 (16.6%) with shoulder pain, 82 (16.2%) with knee pain, 42 (8.3%) with ankle pain, and 21 (4.1%) with hand pain. Of all the participants, 333 (65.9%) were women with mean ± standard deviation (SD) age of 52.4 ± 15.1 years and mean ± SD pain duration of 22.6 ± 57.4 weeks.

**Table 1:** Demographic information.

Characteristics	Mean (SD) or N (%)	Range
Gender (female)	333 (65.9%)	
Age (years)	52.4 (15.1) 20–80	20–80
Height (cm)	161.7 (8.7)	137–191
Weight (kg)	59.5 (12.3)	35–118
Duration of pain (week)	22.6 (57.4)	1–528

Ethical approval was obtained from the Institutional Ethics Committee of Konan Woman's University. Written informed consent was obtained from all the participants before the study, and the study was conducted in accordance with the tenets of the Declaration of Helsinki.

### Procedures

All participants were assessed for demographic data (age, gendar, height, and weight), pain duration, CSI, health-related quality of life (QOL), pain intensity, and pain interference. CSI-J consists of Parts A and B. Part A consists of a 25-item self-report questionnaire designed to assess health-related symptoms that are common to CSS. Each item is rated on a 5-point Likert-type scale, with total scores ranging from 0 to 100. Part B (which is not scored) asks the participants whether one or more specific disorders, including seven separate CSSs, have been diagnosed previously (restless leg syndrome, chronic fatigue syndrome, fibromyalgia, temporomandibular joint disorder, migraine or tension headaches, irritable bowel syndrome, multiple chemical sensitivities, neck injury (including whiplash], anxiety or panic attacks, and depression).

Health-related QOL was measured using the EuroQol 5-dimension (EQ-5D) instrument [39]. EQ-5D was developed as a non-disease specific standardized instrument, which could be used to complement existing health-related QOL measure [40, 41]. It comprises five dimensions: mobility, self-care, usual activities, pain/discomfort, and anxiety/depression. Each dimension has three grades (no problems, some problems, and extreme problems), which generates a single index value for each health state. These values are numbers on a scale where 1 refers to full health and 0 refers to death.

Pain intensity and interference were measured using the Brief Pain Inventory (BPI) [42, 43]. BPI comprises four pain intensity and seven pain interference items. These items are rated using a scale of 0–10, where 0 = no pain and 10 = worst possible pain. Based on the values obtained, individual pain intensity and interference scores were evaluated by calculating the mean. The validation and clinical utility of BPI has been evaluated for several disorders [44–46].

### Development of a short version of CSI

#### Rasch analysis

The original CSI was consecutively analyzed using the Rasch model by Winsteps software (v3.90.2, Chicago, Illinois). Chi-square fit statistics were used to determine how well each CSI item contributed to total CSI scores [47]. The most commonly used chi-squares are known as outfit and infit, which are reported as mean squares (in logits) [48]. Rasch analysis examines the predictability of the data when assessing item quality directly using the mean-square statistics. Excessively large-fit residuals (>1.3 logits) indicate a large difference between the expected and observed performance of an item [47], and may indicate that the item is assessing a construct other than what it was intended to assess. In contrast, excessively small-fit residuals (<0.7 logits) indicate items that behave too predictably [48]. Successive Rasch analyses were performed until a final set of items satisfied the model fit requirements.

#### Psychometric properties of a short version of CSI

We investigated the psychometric properties of the original and shortened questionnaires, and compared the following components:

#### Category order

We assessed category order to ensure the Likert scale functioned as expected. The CSI has five response categories (0 = Never, 1 = Rarely, 2 = Sometimes, 3 = Often, 4 = Always). Category probability curves, and average measure and category fit statistics (infit and outfit) were used to explore rating scale functioning. Fit statistics are recommended to be between 0.6 and 1.4[49, 50]. Moreover, in a well-functioning rating scale, each curve has a distinct peak and 4 clear thresholds that represent the point at which the likelihood of endorsing one category is equal to that of endorsing the next. Disordered thresholds can occur if a category is underutilized or respondents use the categories in an unexpected manner (e.g., respondents cannot differentiate between the categories). In addition, the item characteristic curve (ICC) was plotted. The item is more discriminating where the ICC is steeper, and less discriminating where the ICC is flatter.

#### Targeting

Scale-to-sample targeting was made by comparing person locations from the sample with item locations of the scale. Their means should be close to 0 logits. In addition, we investigated targeting by visualizing the person-item map and comparing the means of the items and person measures. This was analyzed by plotting the person-item location threshold distribution graph with distributions of persons on the top half of the graph and item thresholds at the bottom half of the graph. Good targeting means that the distributions of persons and distributions of item thresholds match visually.

#### Internal consistency

Winsteps provides a person reliability index and Cronbach’s alpha as indicators of internal consistency [51] and both should exceed 0.7 [52]. McDonald’s Omega was also calculated using Mplus 8 (Los Angeles, CA, United States).

#### Unidimensionality

We intended to summate the CSI to provide an overall measure of CS-related symptomology. Individual items should share this unidimensional construct yet be sufficiently different to warrant their inclusion. Assessment of fit evaluates unidimensionality by identifying items that function unexpectedly. The principal component analysis of residuals (PCA) identifies unexpected clusters of items suggestive of a secondary dimension that could threaten measurement of the primary dimension. The PCA residual correlation matrix was visually inspected to identify clusters of items that would be suggestive of a second dimension. An eigenvalue greater than 2.0 for the PCA of residuals suggests a second dimension [53]. Response dependency between the items was examined by inspecting the residual correlation matrix [52] for pairs of items with correlations exceeding 0.4 [54–55].

The dimensionality of both versions was also explored using exploratory structural equation modeling (ESEM) with geomin oblique rotation in Mplus 8. We tested various models: from a one-factor model to a five-factor model in the CFI-25 and from a one-factor model to a two-factor model in the CFI-9. A minimum cutoff of 0.95 for Comparative Fit Index (CFI), a maximum cutoff of .08 for Root Mean Square Error of Approximation (RMSEA), and a maximum cutoff of 0.08 for Standardized Root Mean Square Residual (SRMR) were considered as indicative of acceptable fit [56–58]. Models with smaller values of Akaike information criterion (AIC) and Bayesian information criterion (BIC) are preferred to those with higher AIC and BIC values.

#### Person fit

Participants with outfit residuals greater than 1.5 logits were examined to determine the reason for poor fit. They were compared with those who fit the model using Fisher’s exact test [59] of significance (for gender) or the Mann-Whitney U test (for age, pain intensity, pain duration, and CSI). Response strings of misfitting participants were analyzed to identify patterns in their responses.

#### Differential item functioning (DIF)

Items should function in a similar manner for all people of similar levels of agreeability. We assessed for DIF across 5 subgroups: gender, age (≤60, >60 years), pain intensity from BPI (≤5, >5), pain duration (≤12, >12 month), and pain interference from BPI (median split: ≤2.75, >2.75). DIF was tested using a Mantel-Haenszel chi-square test with significance level set at p = 0.01 for each item. Item bias was explored if an item yielded a significant difference of greater than 0.5 logits between subgroups [60].

#### Test-retest reliability

CSI reliability was assessed using scores obtained from a second round of the questionnaire administered to participants within 2 weeks of the first questionnaire completion. An intraclass correlation coefficient (ICC) 2-way mixed model with absolute agreement was used to determine measurement reliability. ICC_3,1_ values 0–0.40 were considered to indicate fair reliability, 0.41–0.60 moderate reliability, 0.61–0.80 substantial reliability, and 0.81–1.00 almost perfect reliability [61].

#### Relationship to clinical status

Correlation analysis was evaluated using the Statistical Package for Social Sciences Version 25 (IBM SPSS Statistics for Windows, Version 25.0. Armonk, NY: IBM Corp.). Data distribution was tested for homoscedasticity using the Kolmogorov-Smirnov test. A series of correlations was performed examining the relationships between the original and the short form of CSI total score and pain intensity, interference, and QOL. These correlations were investigated with Spearman’s correlation coefficient.

In addition, the correlation between the original and the short form of CSI total score was investigated by calculating the ICC_3,1_ and the 95% confidence interval for agreement. ICC_3,1_ values 0–0.40 were considered to indicate fair reliability, 0.41–0.60 moderate reliability, 0.61–0.80 substantial reliability, and 0.81–1.00 almost perfect reliability [61].

#### Short form of CSI total score groupings by CSS diagnoses

Five severity levels of the original CSI (subclinical = 0–29, mild = 30–39, moderate = 40–49, severe = 50–59, and severe = 60–100) were recommended to evaluate symptom severity or assess clinical changes in response to treatment [58]. To determine the level of CSI severity in the shorter version of CSI, the difference in shorter version of CSI scores was compared with the number of CSS (0 vs. 1 vs. 2 vs. 3+) using one-way analysis of variance (ANOVA) and Tukey post-hoc analysis as well as the original CSI did [62]. CSS diagnoses were determined from subject self-report on CSI part B. P values of <0.05 were considered statistically significant.

The differences in age, pain duration, pain intensity, pain interference, and health-related QOL were also compared with the shortened CSI severity level group using ANOVA and Tukey post-hoc analysis. Gender was compared with the shortened CSI severity level group using the chi-squared test. We adjusted alpha to 0.008 because we undertook six separate analyses.

## Results

Table 2 provides a summary of the clinical profile of all participants. Of 505 participants, 377 (74.6%) participants had acute pain and 128 (25.4%) participants had chronic pain.

**Table 2:** Clinical profiles.

Clinical profiles	Mean (SD)	Range
Pain intensity (BPI, 0–10)	2.9 (1.7)	0–9.75
Pain interference (BPI, 0–10)	2.5 (2.1)	0–10.0
Health-related QOL (EQ-5D, 0–1)	0.71 (0.11)	0.334–1
Central sensitization (CSI-25, 0–100)	21.4 (12.9)	0–80
Central sensitization (CSI-9, 0–36)	10.9 (5.8)	0–32

EQ-5D: EuroQol 5-dimension, CSI: Central sensitization inventory, BPI: Brief Pain Inventory.

### Development of the shortened questionnaire

Four consecutive Rasch analyses identified the items that could be removed from the questionnaire (Table 3). Finally, the shortest questionnaire that maintained correct psychometric properties based on the Rasch model was obtained. It contained only 9 items (CSI-9). The CSI-9 items include: 1. Unrefreshed in morning; 2. Muscles stiff/achy; 3. Pain all over body; 4. Headaches; 5. Do not sleep well; 6. Difficulty concentrating; 7. Stress makes symptoms worse; 8. Tension in neck and shoulders; 9. Poor memory. (S1 Table). Table 4 summarizes the fit statistics for the original CSI (CSI-25) and CSI-9.

**Table 3:** Item selection from Rasch analysis.

	CSI-25	1^st^ Round	2^nd^ Round	3^rd^ Round	4^th^ Round Final set
Item removed	-	4, 5, 7, 8, 16, 17, 19, 20, 24, 25	6, 11, 14	3, 21	22
Item remaining	1, 2, 3, 4, 5, 6, 7, 8, 9, 10, 11, 12, 13, 14, 15, 16, 17 18, 19, 20, 21, 22, 23, 24, 25	1, 2, 3, 6, 9, 10, 11, 12, 13, 14, 15, 18, 21, 22, 23	1, 2, 3, 9, 10, 12, 13, 15, 18, 21, 22, 23	1, 2, 9, 10, 12, 13, 15, 18, 22, 23	1, 2, 9, 10, 12, 13, 15, 18, 23
Emotional distress	15, 16, 17, 24	15	15	15	15
Urological and general symptoms	9, 11, 21, 22, 23, 25	9, 11, 21, 22, 23	9, 21, 22, 23	9, 22, 23	9, 23
Muscle problem	2, 18	2, 18	2, 18	2, 18	2, 18
Headache/jaw symptoms	4, 10, 19	10	10	10	10
Sleep disturbance	1, 8, 12	1, 12	1, 12	1, 12	1, 12
Not loading	3, 6, 7, 8, 13, 14, 20	3, 6, 13, 14	3, 13	13	13

Information about the number of items addressing each content in the CSI-25 and CSI-9

**Table 4:** Average item endorsability thresholds, including fit statistics.

	Original (25- item)				Short form (9-item)			
Item	Measure (Logits)	SE	Infit (mnsq)	Outfit (mnsq)	Measure (Logits)	SE	Infit (mnsq)	Outfit (mnsq)
2	-1.53	0.04	0.94	1.03	-1.27	0.05	0.97	1.01
18	-1.46	0.04	1.19	1.20	-1.18	0.05	1.14	1.12
1	-1.10	0.05	0.72	0.72	-0.73	0.05	0.70	0.71
12	-0.50	0.05	0.85	0.84	0.03	0.06	0.94	0.91
8	-0.50	0.05	**0.67**	**0.65**	-	-	-	-
25	-0.45	0.05	**1.40**	**1.43**	-	-	-	-
5	-0.41	0.05	**1.35**	**1.53**	-	-	-	-
14	0–0.31	0.05	**1.26**	**1.26**	-	-	-	-
10	-0.23	0.05	**0.94**	**0.98**	0.38	0.06	1.05	1.07
17	-0.08	0.05	**0.61**	**0.59**	-	-	-	-
13	-0.04	0.05	**0.72**	**0.66**	0.60	0.06	0.94	0.84
4	-0.04	0.05	**1.37**	**1.45**	-	-	-	-
15	-0.04	0.05	**0.78**	**0.73**	0.61	0.06	1.01	0.98
9	-0.02	0.05	**1.00**	**0.93**	0.64	0.06	1.15	1.05
22	0.01	0.06	**1.15**	**1.00**	-	-	-	-
16	0.14	0.06	**0.65**	**0.57**	-	-	-	-
21	0.18	0.06	**1.27**	**1.27**	-	-	-	-
23	0.21	0.06	**0.97**	**0.98**	0.92	0.07	1.26	1.29
20	0.51	0.07	**1.41**	**1.25**	-	-	-	-
7	0.52	0.07	**1.33**	**1.18**	-	-	-	-
3	0.70	0.07	**1.22**	**0.97**	-	-	-	-
19	0.79	0.07	**1.40**	**1.10**	-	-	-	-
6	0.96	0.08	**1.29**	**1.25**	-	-	-	-
11	1.13	0.09	**1.29**	**1.03**	-	-	-	-
24	1.57	0.11	**1.37**	**0.93**	-	-	-	-

Bold type indicates excessive item misfit.

Negative item measures indicate items that are easier to endorse, and positive measures indicate items that are more difficult to endorse. SE = standard error of measure, mnsq = mean square (chi-square based fit statistic).

### Psychometric assessment of CSI

#### Targeting

**Fig 1:**
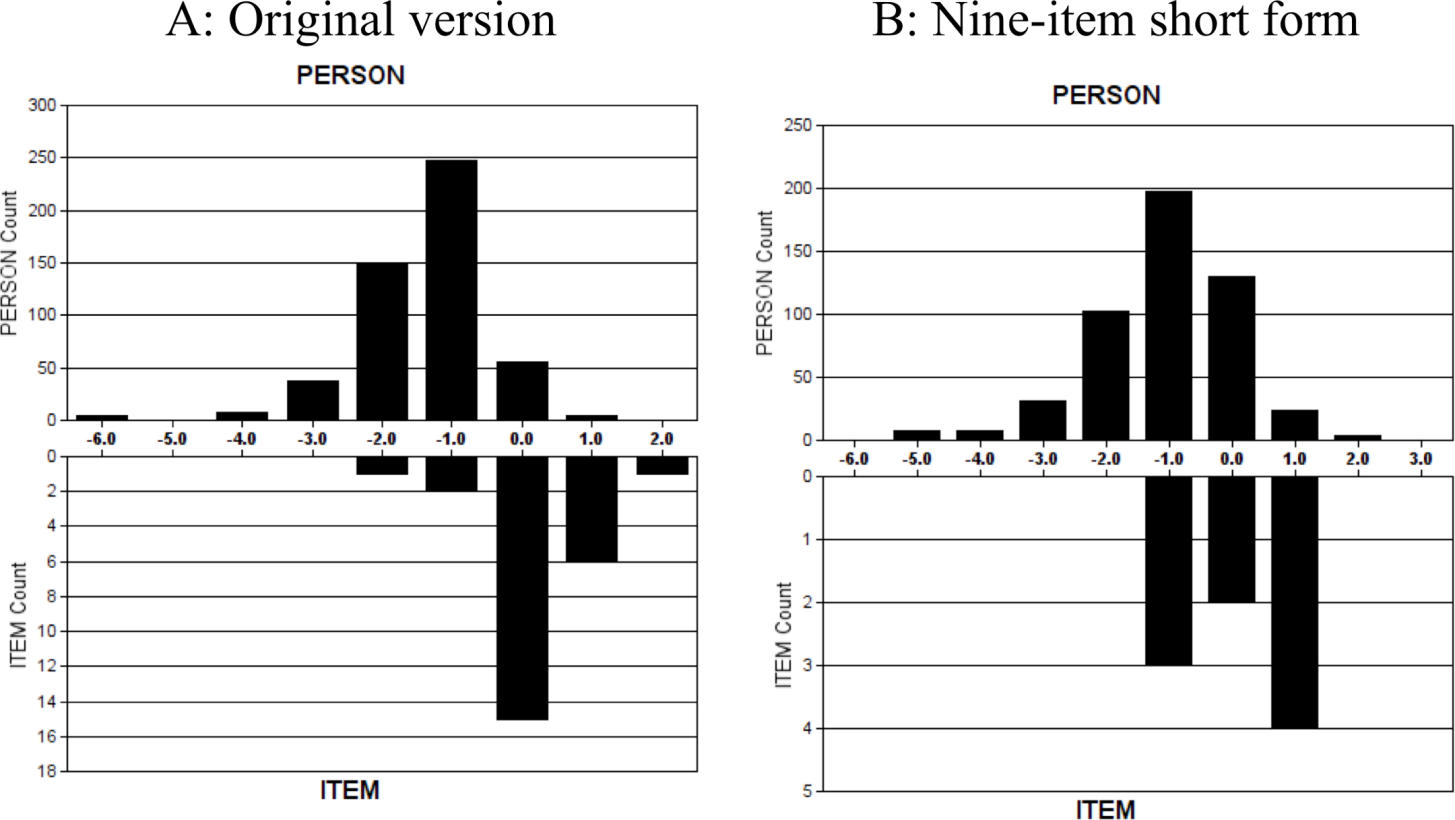
Item–person threshold map. A. Original version. B. Nine-item short form. Persons of lesser central sensitization and easier items are located on the left side of the logit scale (i.e. < 0 logits); persons of higher central sensitization and greater difficulty items are located to the right of the logit scale (i.e. > 0 logits). Item endorsability mean is set at 0 logits by default.

The sample was not well targeted by both versions of CSI. The average person endorsability of CSI-25 and CSI-9 (mean ± SD logits) were -1.42 ± 0.91 and -1.09 ± 1.11, respectively. The item endorsability averages of CSI-25 and CSI-9 (mean ± SD logits) were 0 ± 0.72 and 0 ± 0.79, respectively. Visually, the shifting of person agreeability of CSI-25 and CSI-9 to the left when compared with item endorsability indicated that participants with low levels of central sensitization were not well targeted by the scale (Fig 1).

In CSI-25 and CSI-9, 4 (0.7%) and 7 participants (1.3%), respectively, scored zero for all the items. None of the participants for CSI-25 and CSI-9 scored full points on all items.

#### Category order

The average agreeability measures of the respondents advanced as expected across the rating scale categories in both CSI-25 and CSI-9, and there was neither excessive positive nor negative fit statistics in CSI-9, suggesting the category structure is adequate, but, in CSI-25, there was no excessive outfit statistics (Table 5).

**Table 5:** Average category score thresholds, including fit statistics.

Category score	CSI-25			CSI-9		
	Measure (Logits)	Infit	Outfit	Measure (Logits)	Infit	Outfit
0	-2.00	0.95	0.97	-2.03	1.06	1.04
1	-1.13	0.96	0.75	-1.17	0.90	0.88
2	-0.58	0.94	0.93	-0.38	0.94	0.96
3	-0.16	1.11	1.26	0.34	0.94	0.95
4	0.20	1.35	**1.65**	0.87	1.16	1.23

Negative item measures indicate items that are easier to endorse, and positive measures indicate items that are more difficult to endorse.

**Fig 2:**
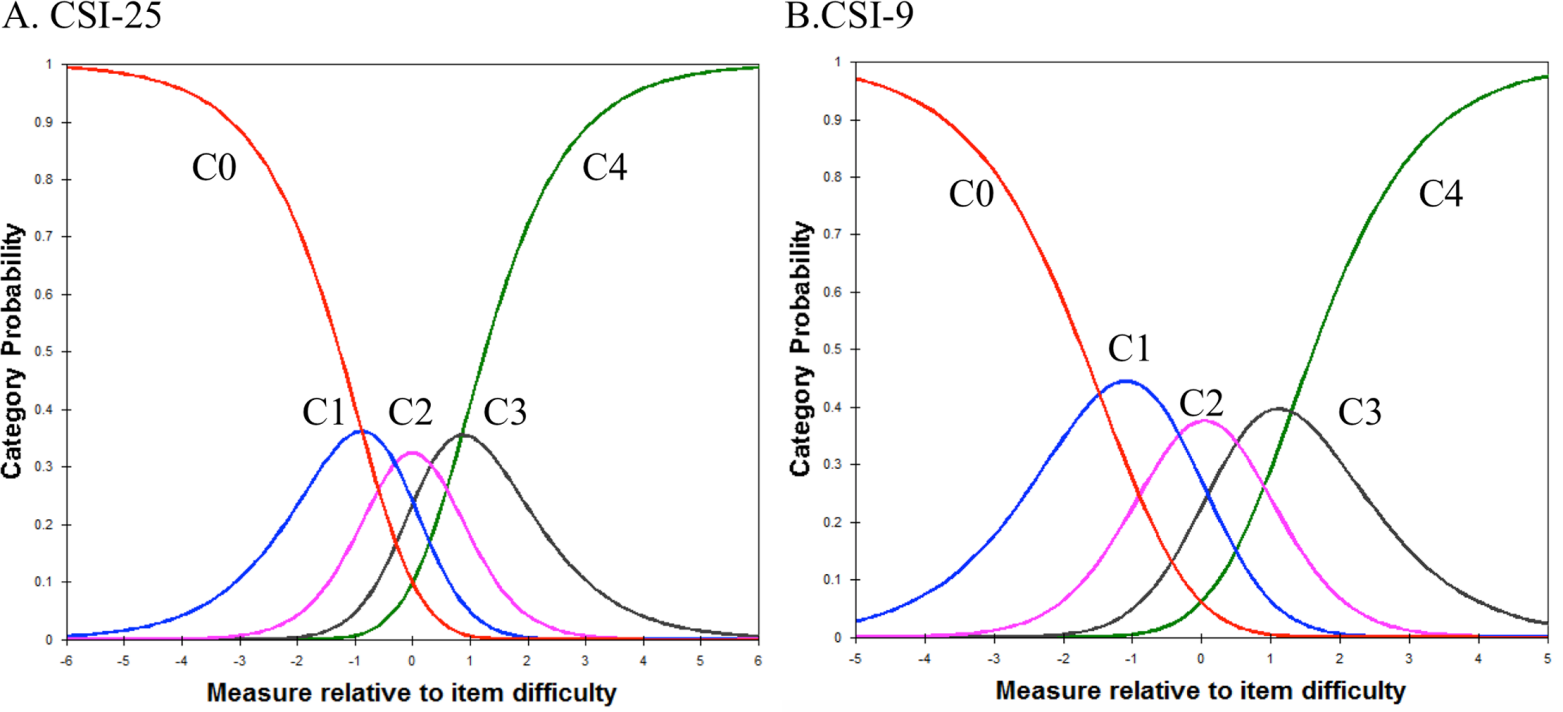
Central sensitization inventory. C0, never; C1, rarely; C2, sometimes; C3, often; C4, always). a = CSI-25, b = CSI-9.

**Fig 3:**
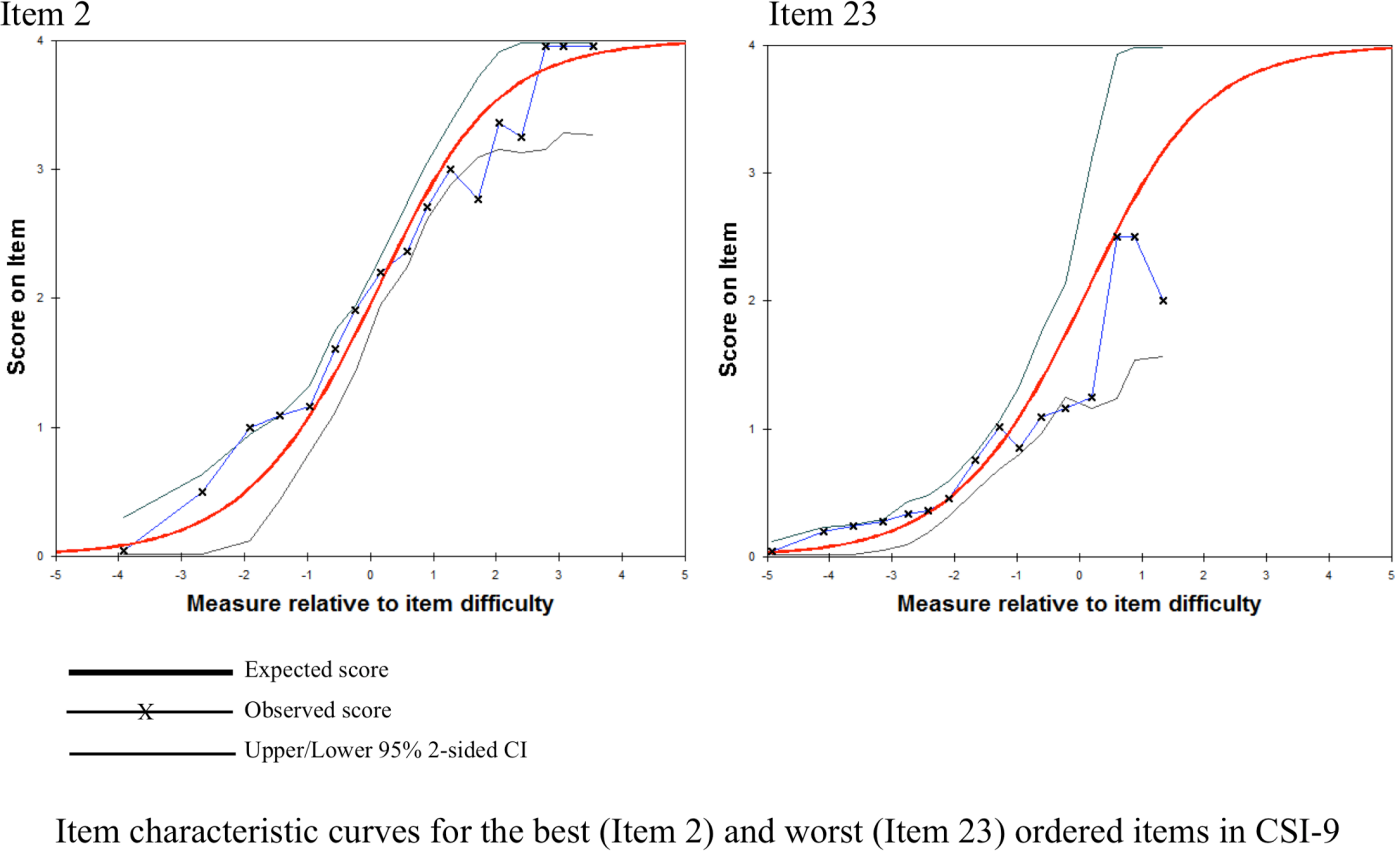
Item characteristic curves for the best (Item 2) and worst (Item 23) ordered items in CSI-9.

Category 4 in CSI-25 was underutilized, which suggests the respondents experienced difficulty differentiating “often” from “always” (Fig 2A). Each category in CSI-9 has a distinct peak suggesting the categories are not disordered. That is, the step calibrations (the thresholds between categories) are ordered as expected (Fig 2B). Fig 3 demonstrated ICC of the best (item 2) and worst (item 23) ordered items in CSI-9.

#### Unidimensionality

PCA of the residuals indicated that the unexplained variance of the first contrast of CSI-25 and CSI-9 was 2.2 and 1.6 eigenvalue units, respectively, indicating multidimensionality of CSI-25 and unidimensionality of CSI-9. The tested five-factor ESEM models in CSI-25 and two-factor model in the CFI-9 were the most fitted to the data (Table 6).

**Table 6:** Exploratory factor analyses using ESEM.

		CFI	SRMR	AIC	BIC	RMSEA
CSI-25	One-factor	0.796	0.057	31501.483	31818.325	0.073
	Two-factor	0.864	0.048	31278.427	31696.658	0.063
	Three-factor	0.914	0.037	31118.771	31634.167	0.052
	Four-factor	0.940	0.031	31043.022	31651.358	0.046
	Five-factor	0.952	0.028	31021.255	31718.307	0.043
CSI-9	One-factor	0.868	0.056	12264.962	12379.025	0.102
	Two-factor	0.942	0.034	12192.360	12340.220	0.081

ESEM: Exploratory Structural Equation Modeling, CFI: Comparative Fit Index, RMSEA: Root Mean Square Error of Approximation, SRMR: Standardized Root Mean Square Residual, AIC: Akaike Information Criterion, BIC: Bayesian information criterion

#### Internal consistency

The person reliability of CSI-25 and CSI-9 was 0.90 and 0.77, respectively. Cronbach’s alpha of CSI-25 and CSI-9 were 0.89 and 0.80, respectively. McDonald’s Omega of CSI-25 and CSI-9 were also 0.89 and 0.80, respectively. These results suggest that both versions of CSI have good internal consistency.

Analysis of person fit of CSI-25 and CSI-9 identified 73 participants (14.4%) and 70 participants (13.8%) with excessive positive outfit (>1.5 logits), respectively. In CSI-25, the CSI-25 score of participants with misfit were significantly higher than those without misfit (mean difference 5.4 point, p < 0.01).

#### Differential item functioning (DIF)

There was differential item functioning for age (items 10, 21, 22m and 23) in CSI-25, but there was no differential item functioning for age, gender, pain intensity, pain interference or pain duration in CSI-9.

#### Test-retest reliability

Of the participants who reported no change (the difference between first and second round of pain intensity during movement is under 10 mm) in pain intensity during the past 2 weeks (n = 100), in CSI-25 and CSI-9, there was excellent agreement between test and retest total scores, with an ICC_3,1_ of 0.86 (95% confidence interval [CI], 0.80–0.9) and 0.79 (95% CI, 0.71–0.85), respectively.

#### Relationship to clinical status

The CSI-9 was significantly correlated with CSI-25 (rho = 0.91, p < 0.001) (Table 7). The CSI-25 and CSI-9 were significantly correlated with pain intensity (0.42 and 0.45, respectively, p < 0.001), pain interference (0.51 and 0.50, p < 0.001), and EQ-5D (-0.43 and -0.41, p < 0.001). The ICC_3,1_ of the CSI-25 and CSI-9 was 0.69 (95% confidence interval, 0.64–0.73), suggesting substantial agreement.

**Table 7:** Correlation between the original CSI, the short form of the CSI, and the clinical variables.

	Original (CSI-25)	Short form (CSI-9)
Original (25-item)	-	0.91*
Pain intensity	0.42*	0.45*
Pain interference	0.51*	0.50*
EQ-5D	-0.43*	-0.41*

* Indicates p < 0.001

#### Short form of CSI total score groupings by CSS diagnoses

Of the 505 participants, 372 (73.6%) reported 0, 96 (19.0%) reported 1, 24 (4.7%) reported 2, and 13 (2.5%) reported ≥ 3 CSS diagnoses. The CSI-9 mean score was 9.9 ± 5.5 in the no CSS group, 13.0 ± 5.0 in the 1 CSS group, 13.7 ± 6.5 in the 2 CSS group, and 20.5 ± 5.7 in the 3 or more CSS group. The CSI score in patients with no CSS was significantly lower than in those with 1, 2, and 3 or more CSS. There was no significant difference in CSI-9 total score between the 1 and 2 CSS groups.

ANOVA identified that there was consistent worsening in pain intensity, pain interference, and health-related QOL with respect to the CSI-9 severity levels (Table 8). The number of men was significantly higher in the subclinical level than that of women, whereas the number of women was significantly higher in the mild level than that of men.

**Table 8:** Comparisons between clinical variables grouped based on the CSI-9 severity.

	Subclinical^1^ 0–9 N = 223	Mild^2^ 10–19 N = 240	Moderate/Severe^3^ 20–36 N = 42	ANOVA or Chi-squared P-value	Effect size
Age	52.8 (15.6)	53.1 (14.6)	46.4 (13.6)	0.02	0.15
Female (%)	129 (57.8) *	174 (72.5) †	30 (71.4)	0.003	0.14
Pain duration (weeks)	15.2 (35.1) ^3^	24.4 (56.8)	51 (118.3) ^1^	0.001	0.16
Pain intensity	2.3 (1.4) ^2,3^	3.3 (1.7) ^1,3^	4.4 (1.9) ^1,2^	< 0.001	0.40
Pain interference	1.6 (1.5) ^2,3^	2.9 (2.1) ^1,3^	4.8 (2.4) ^1,2^	< 0.001	0.49
EQ-5D	0.74 (0.11) ^2,3^	0.70 (0.11) ^1,3^	0.60 (0.11) ^1,2^	< 0.001	0.35

Post-hoc differences between severity-level groups are indicated via superscript number.

* Male > Female

†Female > Male

Standard deviations or percentage female in parentheses

### Discussion

The current study aimed to develop a short form of CSI and evaluate its psychometric properties. This is the first study to develop and validate a shortened CSI. The shortened CSI was obtained by reduction of the long and well-validated questionnaires by removing selected items using Rasch analysis. Rasch analysis has been proposed as an objective and optimal method to construct measurement scales and has recently been used to reduce the number of items in other questionnaires. In the present study, CSI-9 had acceptable internal consistency, unidimensional attributes, no notable DIF, test–retest reliability, and was functional on the category rating scale. Overall, CSI-9 showed adequate psychometric properties for use in evaluating health symptoms related to CSS in patients with musculoskeletal pain disorders. Therefore, CSI-9 might be useful in treatment planning when a shortened version of a questionnaire is needed, such as in a time-limited situation or for a specific population.

As result of Rasch analysis, 9 items were selected. The face validity of the CSI-9 items is very good. For instance, the items correspond very well with the current criteria for fibromyalgia, using the Widespread Pain Index [63] and Symptom Severity Score, which assesses widespread pain distribution, fatigue, unrefreshed sleep, and cognitive symptoms.

The average person endorsability of CSI-25 and CSI-9 were *-*1.42 logits and -1.09 logits, respectively. Both versions covered average and high levels of somatic and emotional symptoms related to central CSS and are therefore best suited for use with people who have health symptoms related to CSS.

Rating categories complied with the set criteria for category functioning and step measures endorsed monotonically from easy to hard across category responses, supporting proper category order in CSI-9.

Although PCA analysis of the residuals of CSI-25 suggests the presence of a second dimension (eigenvalue > 2.0), CSI-9 has unidimensional attributes (eigenvalue < 2.0). This result supports the notion that CSI-9 is appropriate with respect to Rasch analysis. Therefore, a meaningful comparison of CS across patients should be conducted using CSI-9.

The internal consistency of CSI-9 was noteworthy (Cronbach’s alpha of 0.80) and aligned well with the original English version (Cronbach’s alpha of 0.87) [7] and Japanese version (Cronbach’s alpha of 0.89). In general, as the number of items in the questionnaire increases, so does the superiority of the value of Cronbach’s alpha. Therefore, Cronbach’s alpha of CSI-9 is lower than that of CSI-25. In addition, the ICC score was 0.79 (95% CI 0.71–0.85), indicating that CSI-9 had excellent reliability.

CSI-9 adequately comprises all five components of CSS (emotional distress = item 15, urological and general symptom = items 9 and 23, muscle symptom = items 2 and 18, headache/jaw symptoms = item 10, sleep disturbance = items 1 and 12, and not loading = item 13) in the Japanese version [21]. Therefore, CSI-9 could cover a wide range of CSS in the Japanese version.

For a satisfactory short form, the reduction in the correlation scores of the short form should not drop by more than 0.10 from the original version [24]. Our results met this requirement by showing equivalence between CSI-25 and CSI-9 scores. In addition, the ICC_3,1_ of the CSI-25 and CSI-9 was 0.69, suggesting substantial agreement, thereby making CSI-9 a promising measure.

In the present study, patients were divided into subgroups according to the number of CSS diagnosis. The CSI-9 total score in patients with no CSS was lower than that in patients with 1, 2, and 3 or more CSS, but there was no difference in the CSI-9 total score between participants with 1 and 2 CSS. Therefore, we determined three levels of severity in CSI-9, which included subclinical (0–9), mild (10–19), and moderate/severe (20–36). The study results suggest that a strong relationship exists between the CSI severity groups and pain intensity, disability, and health-related QOL. The lowest CSI severity group reported lower pain intensity, disability, and health-related QOL, whereas the highest CSI severity group reported higher pain intensity, disability, and health-related QOL. These results suggest that the CSI severity groups in CSI-9 have high concurrent and construct validity. Therefore, clinically relevant symptom severity levels provide more useful information to healthcare providers in assessing CS/CSS-related symptoms.

Our results are not intended to suggest abandoning CSI-25. CSI-25 (original version) has been psychometrically validated in many languages, and its dimensionality and reliability have been established, suggesting that CSI could detect comprehensive traits of CS. The CSI-9 would be a valid and reliable option if researchers or clinicians want to use an abbreviated measure of CSS-related symptoms, such as screening to rule out a disorder or epidemiological surveys.

There were several limitations to our study. First, our study included individuals between the age of 20 and 80 years. Therefore, it is possible that age, sex, pain intensity, and pain duration influenced the results of psychometric properties. However, a notable DIF was not found for any of the items in CSI-9, suggesting that there is no effect of participant age, sex, or pain intensity and duration. This finding indicates that these factors might not affect the model. Second, this study included a convenience sample of participants with musculoskeletal disorders who were recruited from a single clinic. Future studies, in which data is compared across multiple centers, are warranted to verify the findings of this study. Third, this study utilized a questionnaire that was psychometrically validated in a Japanese sample and therefore validation of the other language version of the short form of CSI is needed. Fourth, the short form of CSI was extracted from one administration of the full CSI. Further research to investigate the specific CSI short form might be required for more definitive conclusions. Fifth, CSS diagnoses were established from the same self-reported instrument as the CSI scores. This procedure is limited as a validation, similar to internal measure based on two different procedures (categorical vs quantitative). Absence of a non-self-reported type and number of clinical diagnosis of CSS is needed in the further study.

### Conclusion

A nine-item short form of CSI has acceptable psychometric properties and is suitable for use for patients with musculoskeletal pain. Thus, CSI-9 can be used as a brief instrument to evaluate CS.

## Supporting information

S1 Table CSI-25 and CSI-9. (DOCX)

S1 Data Anonymized data. (ZIP)
