# Supplementary material for: Development and psychometric properties of short form of central sensitization inventory in participants with musculoskeletal pain: A cross-sectional study
Source: PLoS One. 2018 Jul 5;13(7):e0200152. doi: 10.1371/journal.pone.0200152 (PMC6033441; doi:10.1371/journal.pone.0200152)
Supplement: S1 Table — (DOCX) [file pone.0200152.s001.docx]

| CSI-25 (Original version) | CSI-9 (Short version) |
| --- | --- |
| 1.Unrefreshed in morning | 1. Unrefreshed in morning |
| 2. Muscles stiff/achy | 2. Muscles stiff/achy |
| 3. Anxiety attacks |  |
| 4. Grind/clench teeth |  |
| 5. Diarrhea/constipation |  |
| 6. Need help with daily activity |  |
| 7. Sensitive to bright light |  |
| 8. Easily tired with physical activity |  |
| 9. Pain all over body | 3. Pain all over body |
| 10. Headaches | 4. Headaches |
| 11. Bladder/urination pain |  |
| 12. Do not sleep well | 5. Do not sleep well |
| 13. Difficulty concentrating | 6. Difficulty concentrating |
| 14. Skin problems |  |
| 15. Stress makes symptoms worse | 7. Stress makes symptoms worse |
| 16. Sad or depressed |  |
| 17. Low energy |  |
| 18. Tension neck and shoulder | 8. Tension neck and shoulder |
| 19. Pain in jaw |  |
| 20. Certain smells make dizzy |  |
| 21. Urinate frequently |  |
| 22. Restless legs |  |
| 23. Poor memory | 9. Poor memory |
| 24. Trauma as a child |  |
| 25. Pelvic pain |  |

Supplement file
